# Analysis of 17,428 pregnant women undergoing non-invasive prenatal testing for fetal chromosome in Northeast China

**DOI:** 10.1097/MD.0000000000024740

**Published:** 2021-02-12

**Authors:** Rulin Dai, Yang Yu, Han Zhang, Leilei Li, Yuting Jiang, Ruizhi Liu, Hongguo Zhang

**Affiliations:** Center of Reproductive Medicine and Center of Prenatal Diagnosis, the First Hospital, Jilin University, Changchun, Jilin, China.

**Keywords:** cell-free DNA, fetal chromosomal abnormality, non-invasive prenatal testing, prenatal screening

## Abstract

Non-invasive prenatal testing (NIPT) is an incomparable prenatal screening technology, but we should undergo amniocentesis to confirm fetal chromosome when pregnancies receive a positive result via NIPT. We aimed to investigate the detection rate and positive predictive value of NIPT results in pregnancies from Northeast China, and to determine the reasons for false positive and false negative NIPT results.

This study evaluates 17,428 singleton pregnancies had undergone NIPT detection. 202 samples were NIPT positive with the detection rate was 1.16% (202/17,428). Among all the positive samples, 160 samples (79.21%) were referred for an amniocentesis procedure to investigate the fetal chromosome. The positive predictive value of T21, T18, and T13 was found to be 75% with a 0.07% false positive rate. Positive predictive value from high to low was as follows: trisomy 21 (84.38%), followed by trisomy 18 (61.54%), autosomal abnormalities (52.94%), sex chromosomal abnormalities (38.46%), and trisomy 13 (33.33%). The positive predictive values for sex chromosome abnormalities turned out to be mosaic sex chromosome aneuploidies (83.33%), followed by XYY (57.14%), XXY (37.50%), XXX (36.36%), and Monosomy X (28.95%). Out of the 160 samples had amniocentesis, the true positive cases in trisomy 21 had a higher percentage of Z-scores compared with the false positive cases in trisomy 21 (*P* < .05). And the true positive cases in trisomy 18 had a significantly higher percentage of Z-scores compared with the false positive cases in trisomy 18 (*P* < .01).

These findings indicate that the positive predictive value of T21, T18, and T13 was found to be 75% with a 0.07% false positive rate. It is worth noting that the positive predictive value of NIPT for autosomes and sex chromosomes. Moreover, if women receive a positive result via NIPT, they should pay attention to the results with undergoing further prenatal diagnosis.

## 1 Introduction

Non-invasive prenatal testing (NIPT) is a milestone in prenatal and fetal field. In 1997, cell free fetal DNA fragments were found in maternal blood,^[1]^ and in 2011, NIPT was applied to clinic.^[2]^ NIPT for fetal aneuploidy using cell-free DNA (cfDNA) has been widely integrated into routine prenatal testing.^[3,4]^ NIPT is a high sensitivity and specificity prenatal screening test for trisomy 21, 18, and 13. Due to the genome-wide properties of NIPT, the scope of screening was widened to include sex chromosome aneuploidies, autosomal trisomies, and sub-microscopic copy number variants.^[5]^

NIPT is an advanced screening method, however, the screening results may differ from the actual fetal karyotypes. The discordant NIPT results can be attributed to several factors. Hartwig et al^[2]^ clarified that confined placental mosaicism, maternal copy number variations (CNVs), maternal malignancy, vanishing twin, and technical, bioinformatics, or human errors were found to be reasons for discordance between NIPT-result and fetal karyotype. Accordingly, a positive NIPT result should always be confirmed by an invasive test like amniocentesis.

With the continually evolving of next-generation sequencing technologies, NIPT also has been applied in several sequencing platforms such as a semiconductor sequencing platform,^[6,7]^ Illumina sequencing platform,^[7,8]^ and the Beijing Genomics Institute (BGI) sequencing platform.^[9]^ We use Illumina sequencing platform for NIPT. One should remember that the NIPT is only a screening test which provides a risk for the genetic disorder, but not the diagnosis. Many companies in the market can now do NIPT testing, and we recommend that pregnancy women should accept it in a qualified prenatal diagnostic center.

Here we aimed to investigate the detection rate and positive predictive value of NIPT results in pregnancies from Northeast China, and to determine the reasons for false positive and false negative NIPT results.

## 2 Subjects and methods

### 2.1 Subjects and study design

We evaluated 17,428 singleton pregnancies had performed NIPT detection who attended the outpatient clinic of the Prenatal Diagnosis Department of the First Hospital of Changchun, Jilin Province, Northeastern China, between July 13, 2017 and January 22, 2020.

We excluded samples presenting clinical indications as follows:

1. Either the woman or her husband had chromosome abnormalities;
2. The woman or her husband had family history of genetic diseases;
3. The woman had structural abnormalities suggested by ultrasound during pregnancy;
4. The woman had malignant tumors during pregnancy.

This study was approved by the ethics committee of the First Hospital of Changchun, Jilin Province (No.2017–452), and all patients provided informed consent to participate in the study.

### 2.2 NIPT analysis

5-ml peripheral blood from individual was collected in EDTA-containing tubes (Sekisui, Tokyo, Japan). The plasma was separated within 72 hours after blood sample collection, maternal peripheral blood (5 ml) was collected and centrifuged for 10 minutes at 4°C at 1600×*g*. The blood cell portion was centrifuged again at 2500×*g* for 10 minutes and the plasma portion at 16,000×*g* for 10 minutes, the blood cells portion and plasma samples were immediately stored at −80°C until further processing.^[10,11]^ cfDNA was isolated with MagMAX Cell-Free DNA Isolation Kit (Applied Biosystems cat.: A29319) according to the manufacture's instruction. DNA was fragmented into an average size of 200 bp. Briefly, 2.5 ng of cfDNA or fragmented DNA was used for the preparation of sequencing libraries. The 8-bp barcoded sequencing adaptors were ligated to fragments and amplified by PCR. Purified libraries were sequenced using NextSeq 550AR (Annoroad Gene Technology Co., Ltd, China). For each maternal plasma sample, an average of 4.2-M reads with 40 bp in length and Q30 > 95% was generated for further analysis.^[12,13]^

Student *t* test was performed based on null/alternative hypotheses, and the relative logarithmic likelihood odds ratio was subsequently calculated. Chromosomal Z-score was calculated using the algorithm described in Qi et al.^[12]^ A Z-score normalization was applied to detect fetal aneuploidy, using the adjusted chromosomal coverage. The calculation accuracy of the Z-score mainly depends on the assumption that there is no mosaic on the fetal aneuploidy chromosomes. Absolute Z-score >3 was used as warning criteria.^[14–16]^

### 2.3 Karyotype analysis of amniotic fluid cells

Amniotic fluid cells were obtained by amniocentesis at 16 to 23 weeks of gestation. They were cultured in CHANG Amnio Medium (Irvine Scientific, Santa Ana, CA), followed by treatment with colcemid. G-banding of metaphase chromosomes was performed by standard methods.^[17]^ For each individual, a minimum of 30 metaphase cells was counted and at least 5 cells were analyzed. Chromosome abnormalities were described according to the criteria established by the International System for Human Cytogenetic Nomenclature.^[17]^ We would recommend that NIPT positive patients to undergo an amniocentesis procedure to investigate the fetal chromosome.

### 2.4 Statistical analysis

The data were compared using the Student *t* test, and analyzed statistically using SPSS software (ver. 17.0; SPSS, Inc., Chicago, IL). Differences were considered to be statistically significant when *P* < .01, with *P* < .05 to indicate statistical significance.

## 3 Results

A total of 17,428 singleton pregnancies had undergone NIPT detection, 202 samples were NIPT positive with the detection rate was 1.16% (202/17, 428). The pregnancy outcomes obtained through follow-up showed that there were no NIPT false negative for the time being. Among all the positive samples, 160 samples (79.21%) were referred for an amniocentesis procedure to investigate the fetal chromosome. The positive predictive value of T21, T18, and T13 was found to be 75% (36/48) with a 0.07% (12/17, 428) false positive rate.

**Figure 1:**
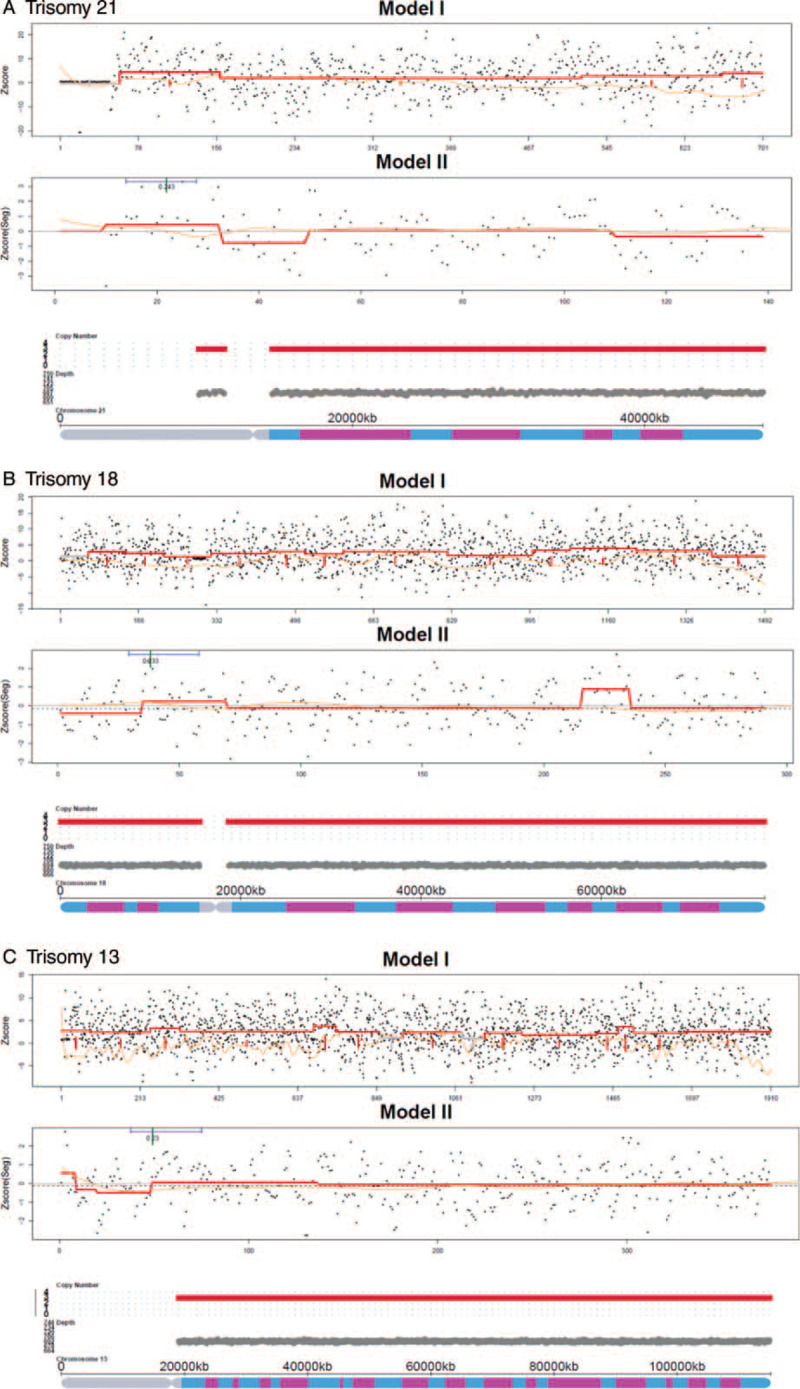
The positive trisomy examples of fetal CNVs detection. A: Trisomy 21. B: Trisomy 18. C: Trisomy 13. Model I: The Z-score distribution map generated by CNV detection is based on Reads count. The black dot (or red dot) indicates that the corresponding Z-score of each alignment window bins. The orange solid line indicates that GC bias of window bins. The blue solid line indicates that Z-score smoothing line is lower than normal. The red solid line indicates Z-score smoothing line is higher than normal. Model II: The Z-score distribution map generated by CNV detection is based on Real unique Reads count. The red line is the Z-score smoothing line generated according to the Z-score of each window bin. The red solid line fluctuates upwards indicates that Z-score is higher than normal. The red solid line fluctuates downwards indicates that Z-score is lower than normal. The 3rd picture is chromosomal diagram generated from Model I and Model II. Red, grey and green bars represent duplication, normal and deletion, respectively. The y-axis shows the chromosomal copy number variations.

In 202 NIPT positive samples, 37 cases (18.32%, 37/202) were predicted to have trisomy 21 and 32 cases (86.49%, 32/37) performed amniocentesis to verify, 27 (84.38%, 27/32) were true trisomy 21. 16 cases (7.92%, 16/202) were predicted to have trisomy 18 and 13 cases (81.25%, 13/16) performed amniocentesis to verify, 8 (61.54%, 8/13) were true trisomy 18. 3 cases (1.49%, 1/202) were predicted to have trisomy 13 and 3 cases (100%, 3/3) performed amniocentesis to verify, 1 (33.33%, 1/3) were true trisomy 13. 55 cases (27.23%, 55/202) were predicted to have autosomal abnormalities except for trisomy 21, trisomy 18 and trisomy 13, and 34 cases (61.82%, 34/55) performed amniocentesis to verify, 18 (52.94%, 18/34) were true abnormalities. Among 91 cases were predicted to have sex chromosomal NIPT positive, 78 cases (85.71%, 78/91) performed amniocentesis to verify, 30 (38.46%, 30/78) were true sex chromosomal abnormalities (Tables 1–3, Fig. 1). Out of the 78 samples opting for amniocentesis, the most frequent true positive rate was mosaic sex chromosome aneuploidies (83.33%, 5/6), followed by XYY (57.14%, 4/7), XXY (37.50%, 6/16), XXX (36.36%, 4/11), and Monosomy X (28.95%, 11/38, Tables 1 and 2). Out of a total of 76 false positive cases in the offline file, maternal copy number variations were present in 6 cases (7.89%, 6/76). Five cases (83.33%, 5/6) were predicted to have sex chromosomal abnormalities, and 1 case (16.67%, 1/6) have autosomal abnormalities.

**Table 1:** Fetal positive results of NIPT.

Fetal karyotype	Detected positive No., n	No. of amniocentesis, n	No. of true positive, n	No. of false positive, n
Trisomy 21	37	32	27	5
Trisomy 18	16	13	8	5
Trisomy 13	3	3	1	2
Sex chromosomal abnormalities	91	78	30	48
Other autosomal abnormalities	55	34	18	16
Total	202	160	84	76

**Table 2:** Sex chromosome positive results of NIPT.

Fetal karyotype	Detected positive No., n	No. of amniocentesis,n	No. of true positive, n	No. of false positive, n
Monosomy X	41	38	11	27
XXX	11	11	4	7
XYY	10	7	4	3
XXY	17	16	6	10
Mosaic sex chromosome aneuploidies	12	6	5	1
Total	91	78	30	48

**Table 3:** Other autosomal abnormalities of NIPT.

Abnormal chromosome	CNVs of NIPT	No.
1	del1q41-1q44(223660001-248360000); del1p34.1-1p32.3(45710001-55810000)	1
2	del2p25.3-2p25.1(3460001-7210000)	1
3	dup3p26.3-3p26.1(260001-4110000)mat	1
3,7	Trisomy 3; Trisomy 7	1
4	del4q22.1-4q22.2(89832601-94432600)mat	1
	del4q32.3-4q34.1(167082601-173182600)mat	1
	del4p15.1-4p14(33607001-37707000)mat	1
	dup4q12-4q13.1(58007701-63157700)mat	1
5	dup5p14.3-5p14.3(19260001-23110000)mat	1
	dup5p14.1-5p13.3(27760001-30860000)mat	1
	Trisomy 5	1
	dup5p14.3-5p13.3(23010001-30110000)mat	1
	del5p15.33-5p15.31(3210001-7060000)	1
6	dup6q12-6q12(65210001-68560000)mat	2
	del6q25.1-6q25.3(151110001-157460000)	1
	del6q27-6q27(165210001-168660000)	1
7	Trisomy 7	3
8	Trisomy 8	2
	del8q23.3-8q23.3(114715101-117315100)	1
	del8p22-8p22(13060001-18160000)	1
9	Trisomy 9	1
	dup9q21.13-9q21.13(74960001-78060000)mat	1
	del9p24.1-9p22.2(6710001-18060000)	1
10	del10q24.32-10q25.2(103110005-112210004)	1
	dup10q11.22-10q11.23(48160001-51960000)mat	3
	del10q11.22-10q11.23(48160001-51960000)mat	1
13	Monosomy 13	1
14	Trisomy 14	1
15	Trisomy 15	3
16	dup16p13.11-16p12.3(15560001-19160000)mat	3
	Trisomy 16	3
	del16p13.13-16p13.11(12060001-16160000)mat	1
18	del18q21.33-18q23(60210001-74360000)	1
	Monosomy 18	1
	dup18q12.2-18q12.3(37060001-40160000)	3
19	dup19p13.2-19p13.2(8310001-12210000)mat	1
21	del21q21.3-21q22.11(30861194-34961193)mat	1
	del21q21.3-21q22.3(30861194-34961193)	1
22	dup22q11.21-22q11.22(18900001-22350004)mat	2
	Trisomy 22	1
Total		55

Del = deletion, Dup = duplication.

**Figure 2:**
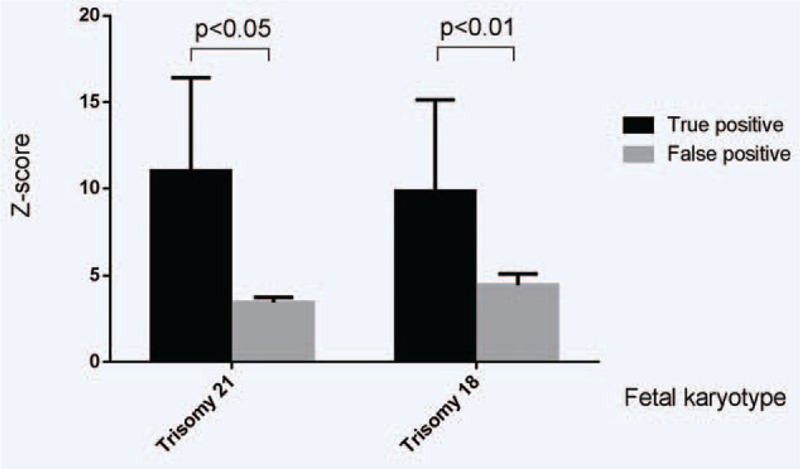
The comparison of Z-scores between true and false positive cases for Trisomy 21 and Trisomy 18.

Out of the 160 samples that had amniocentesis, we identified 5 false positive cases of trisomy 21, the average of Z-scores in trisomy 21 false positive cases was 3.43 ± 0.31. And 27 were true trisomy 21, the average of Z-scores in trisomy 21 true positive cases was 11.02 ± 5.40. The true positive cases in trisomy 21 had a higher percentage of Z-scores compared with the false positive cases in trisomy 21 (*P* < .05). We found 5 false positive cases of trisomy 18, the average of Z-scores in trisomy 18 false positive cases was 4.43 ± 0.66. And 8 were true trisomy 18, the average of Z-scores in trisomy 18 true positive cases was 9.85 ± 5.29. The true positive cases in trisomy 18 had a significantly higher percentage of Z-scores compared with the false positive cases in trisomy 18 (*P* < .01). We found 2 false positives and 1 true positive cases of trisomy 13. The data were too small to complete the statistical analysis (Table 1, Fig. 2).

## 4 Discussion

As NIPT for trisomy 21, 18, and 13 has made big steps in prenatal screening, many pregnant women would undergone NIPT to identify the potential risk of trisomy 21, 18, or 13 for their fetuses. However, false positive and false negative NIPT results were not ignorable.^[2]^ In the current study, we investigated the rates and numbers of NIPT results in pregnancies from Northeast China.

In our study, the NIPT positive with detection rate was 1.16%. The positive predictive value of T21, T18, and T13 was found to be 75% with a 0.07% false positive rate. Positive predictive value from high to low was as follows: trisomy 21 (84.38%), followed by trisomy 18 (61.54%), autosomal abnormalities (52.94%), sex chromosomal abnormalities (38.46%), and trisomy 13 (33.33%). Numerous studies have shown sensitivity rates for NIPT was approximately 99% with false positive rates below 1% and the positive predictive value is limited to 40% to 90%.^[18,19]^ The positive predictive values of NIPT for autosomes and sex chromosomes should be paid attention to. The mosaic sex chromosome aneuploidies included an increase or decrease of sex chromosomal mosaicisms to different degrees, which had the most frequent true positive rate in sex chromosomal abnormalities. Although the lowest frequent true positive rate of sex chromosomal abnormalities was monosomy X (28.95%), but it definitely did not mean that monosomy X could be ignored. Maybe the number of positives in our study was not enough. Similarly, autosomal abnormalities except for trisomy 21, trisomy 18, and trisomy 13 also had this kind of situation. Table 3 shows all the NIPT results suggesting autosomal abnormalities except for trisomy 21/18/13. In our study, abnormal results of NIPT will appear on all chromosomes except for chromosomes 11, 12, 17, and 20. No matter what kind of chromosome abnormality is predicted by NIPT result, it should be verified by amniocentesis.

The calculation accuracy of the Z-score mainly depends on the assumption that there is no mosaic on the fetal aneuploidy chromosomes. We also analyzed the Z-score after the NIPT testing and found that the Z-score of false positive was close to the upper limit of the standard value. Especially, the true positive cases in trisomy 21 and trisomy 18 had a higher percentage of Z-scores compared with the false positive cases, however, the number of true positive and false positive of trisomy 13 was too small to get the conclusion.

Many factors may contribute to false positive and false negative NIPT results, including placental mosaicism, maternal copy number variations, maternal malignancy, vanishing twin, and technical, bioinformatics, or human errors.^[2]^ Maternal duplication could intensify the risk of false-positive results by increasing the number of relative unique mapped chromosome reads and chromosomal coverage, which would lead to a higher Z-score. Maternal deletion would cause the risk of false negatives oppositely. We speculated that the reason for higher false positive rate in detecting sex chromosomal abnormalities from NIPT was maternal CNV, which led to a deviation in the Z-score calculation. Some studies have confirmed that some false-positive results are caused by maternal CNV.^[20–22]^ In our study, 6 false positive cases have maternal CNV, which was one cause of false positive skeptically. The cfDNA in maternal circulation that originates from the pregnancy is derived primarily from placental tissue and may not necessarily represent fetal genetic status.^[23–25]^ cfDNA can get from multiple sources because NIPT can not only reflect the fetal karyotype but also the potential conditions, such as confined placental mosaicism, maternal cancer, or a previously unrecognized maternal genetic condition. However, it is an advanced technique with potential vulnerabilities.^[2]^

NIPT is an incomparable screening test for fetal aneuploidy. If women receive positive results on chromosomal abnormalities via NIPT, they should pay attention to the results and opt for further prenatal diagnosis. NIPT is currently being implemented in the market to detect trisomy 21, trisomy 18, and trisomy 13 with high accuracy. In fact, the positive predictive value of NIPT for autosomes and sex chromosomes cannot be ignored. Although the total number of samples for NIPT is large enough in our study, the number of positives is not enough, and we will continue to collect samples for further study. If we want to clarify the reasons for false positive and false negative NIPT results, the information exchange between clinics and laboratories should be emphasized.

## 5 Conclusion

NIPT is an incomparable prenatal screening technology, but the pregnant women should undergo amniocentesis to confirm fetal chromosome when they receive positive results via NIPT, further genetic counselling should be offered simultaneously. In our study, the positive predictive value of T21, T18, and T13 was found to be 75% with a 0.07% false positive rate. It is worth noting that the positive predictive value of NIPT for autosomes and sex chromosomes.

## Author contributions

**Conceptualization:** Ruizhi Liu.

**Data curation:** Han Zhang.

**Funding acquisition:** Ruizhi Liu.

**Investigation:** Yang Yu, Han Zhang.

**Methodology:** Yang Yu, Leilei Li.

**Software:** Yuting Jiang.

**Writing – original draft:** Rulin Dai.

**Writing – review & editing:** Hongguo Zhang.

## Abbreviations

Abbreviations: cfDNA = cell-free DNA, CNVs = copy number variations, NIPT = Non-invasive prenatal testing.
